# Dentine Regeneration With Calcium Strontium Silicate: In Vitro Odontogenic Differentiation, Antimicrobial Activity, Immunomodulation and In Vivo Pulpotomy in Rat Molars

**DOI:** 10.1111/iej.70165

**Published:** 2026-04-20

**Authors:** Mohamed Mahmoud Abdalla, Vidhyashree Rajasekar, Heba Ahmed, Mengyu Huang, Cynthia Kar Yung Yiu

**Affiliations:** ^1^ Restorative Dental Sciences, Faculty of Dentistry The University of Hong Kong Pokfulam Hong Kong; ^2^ Dental Biomaterials Faculty of Dental Medicine Al‐Azhar University Cairo Egypt; ^3^ Paediatric Dentistry, Faculty of Dentistry The University of Hong Kong Pokfulam Hong Kong; ^4^ General Pathology, Faculty of Medicine Ain‐Shams University Cairo Egypt

**Keywords:** calcium strontium silicate, immunomodulation, odontogenic differentiation, pulpotomy, rat molars, vital pulp therapy

## Abstract

**Aim:**

This study examined the in vitro and in vivo performance of calcium strontium silicate (CSR) as a novel biomaterial for vital pulp therapy (VPT), assessing its odontogenic differentiation, antibacterial activity, immunomodulation, inflammatory response and dentine regeneration potential compared to calcium silicate (CS) and Mineral Trioxide Aggregate (MTA).

**Methodology:**

CSR was synthesized via the sol–gel process. For the in vitro study, human dental pulp stem cells (HDPSCs) were assessed for viability, live/dead staining, trans‐well migration assays and odontogenic gene expression (ALP, DSPP, DMP‐1, RUNX2) using CCK‐8, and RT‐qPCR. Antibacterial activity against *Streptococcus mutans* and *Lactobacillus acidophilus* was assessed via colony‐ forming unit (CFU) counts. Immunomodulation was evaluated by RT‐qPCR for inflammatory cytokines (IL‐1β, TNF‐α, IL‐10). In the in vivo study, inflammatory responses were evaluated in Sprague–Dawley rats subjected to subcutaneous implantation for 7, 14 and 60 days, and pulpotomy procedures were performed on rat maxillary first molars and assessed after 30 and 60 days. The outcomes were analysed using micro‐CT imaging and H&E staining. Statistical analyses included one‐way ANOVA and non‐parametric tests (*p* < 0.05).

**Results:**

CSR significantly enhanced HDPSCs viability, migration and odontogenic gene expression compared to CS and MTA (*p* < 0.05). It exhibited superior antibacterial activity, with the lowest CFU counts (*p* < 0.05). CSR upregulated anti‐inflammatory cytokine IL‐10 and reduced pro‐inflammatory cytokines. In vivo, CSR showed milder inflammation than CS and MTA at 7, 14 and 60 days (*p* < 0.05) and formed consistent calcified bridges in pulpotomy, with no pulp necrosis.

**Conclusions:**

CSR demonstrated superior biocompatibility, regenerative potential, and antibacterial properties making it a promising alternative to MTA for VPT, enhancing clinical outcomes.

## 1 Introduction

In recent years, the field of endodontics has witnessed a remarkable transformation, with a growing emphasis on more regenerative and minimally invasive treatment options to preserve tooth vitality (Duncan et al. 2023). Traditional root canal treatment, although effective in eradicating infection and preserving tooth structure, possesses several drawbacks that can adversely affect the tooth and oral health in the long run (Pak et al. 2012; Peters 2004; Lambrianidis 2006; Lin et al. 2005). Consequently, there is an escalating interest in investigating alternative conservative treatment strategies, such as vital pulp therapy (VPT) which includes direct pulp capping, partial pulpotomy, and complete pulpotomy. These approaches endeavour to preserve the vitality of dental pulp and bolster its innate healing capabilities, culminating in improved patient outcomes (Asgary et al. 2024).

A crucial determinant of success in these minimally invasive procedures is the selection of appropriate pulp capping materials. Currently, Mineral Trioxide Aggregate (MTA) is considered the gold standard for this purpose, owing to its exemplary biocompatibility, sealing capacity and regenerative potential demonstrated in multiple studies (Bogen and Chandler 2008). Nonetheless, MTA is not without its limitations, which include challenging handling properties, extended setting time and the potential for tooth discoloration (Parirokh and Torabinejad 2010). These shortcomings underscore the necessity for developing innovative biomaterials capable of surmounting MTA's limitations while retaining its regenerative prowess.

An optimal material for VPT should not only exhibit superior biocompatibility and sealing capability but also possess attributes that can augment the healing process. These desirable characteristics include the capacity to induce odontogenic differentiation of human dental pulp stem cells (HDPSCs), modulate immune responses and promote macrophage polarization (Ballikaya and Çelebi‐Saltik 2023). Furthermore, the material should demonstrate antimicrobial activity against prevalent caries pathogens, such as *Streptococcus mutans* and *Lactobacillus acidophilus* , which contribute to pulp inflammation and could undermine the success of VPT (Love and Jenkinson 2002).

Emerging research indicates that strontium‐containing materials possess numerous properties that qualify them as promising candidates for VPT applications. Strontium has been reported to promote odontogenic differentiation (Huang et al. 2016), modulate immune responses, and stimulate macrophage polarization (Li et al. 2023). Additionally, strontium‐containing materials have displayed antimicrobial activity against common caries pathogens, further bolstering their potential utility in VPT (Brauer et al. 2013; Abdalla et al. 2023).

In a prior study, calcium strontium silicate (CSR) was successfully developed using the sol–gel process. The synthesized cement exhibited superior mechanical and biological properties compared to calcium silicate (CS), highlighting its potential as a promising material for enhancing the outcomes of regenerative dental treatments (Abdalla et al. 2022). However, a critical evidence gap remains. CSR's capacity to promote dentine regeneration has not been evaluated in *an* in vivo pulpotomy animal model. This gap in preclinical evidence limits validation of efficacy and safety under physiological conditions, underscoring the need for animal studies. In our present study, we extended this research by assessing the in vitro ability of CSR to promote odontogenic differentiation of HDPSCs, inhibit cariogenic bacteria, and modulate the expression of inflammatory cytokines, in comparison with CS and MTA. Additionally, we examined the in vivo inflammatory response to CSR and its potential for dentine bridge formation following pulpotomy in a rat model, comparing these outcomes with CS and MTA. The null hypotheses tested were (i) there are no differences among CSR, CS and MTA in the odontogenic gene expression of HDPSCs, in inhibiting cariogenic bacteria or in modulating the expression of inflammatory cytokines and (ii) there are no differences among CSR, CS and MTA in the in vivo inflammatory response or in their ability to promote dentine bridge formation following pulpotomy in a rat model.

## 2 Materials and Methods

**FIGURE 1:**
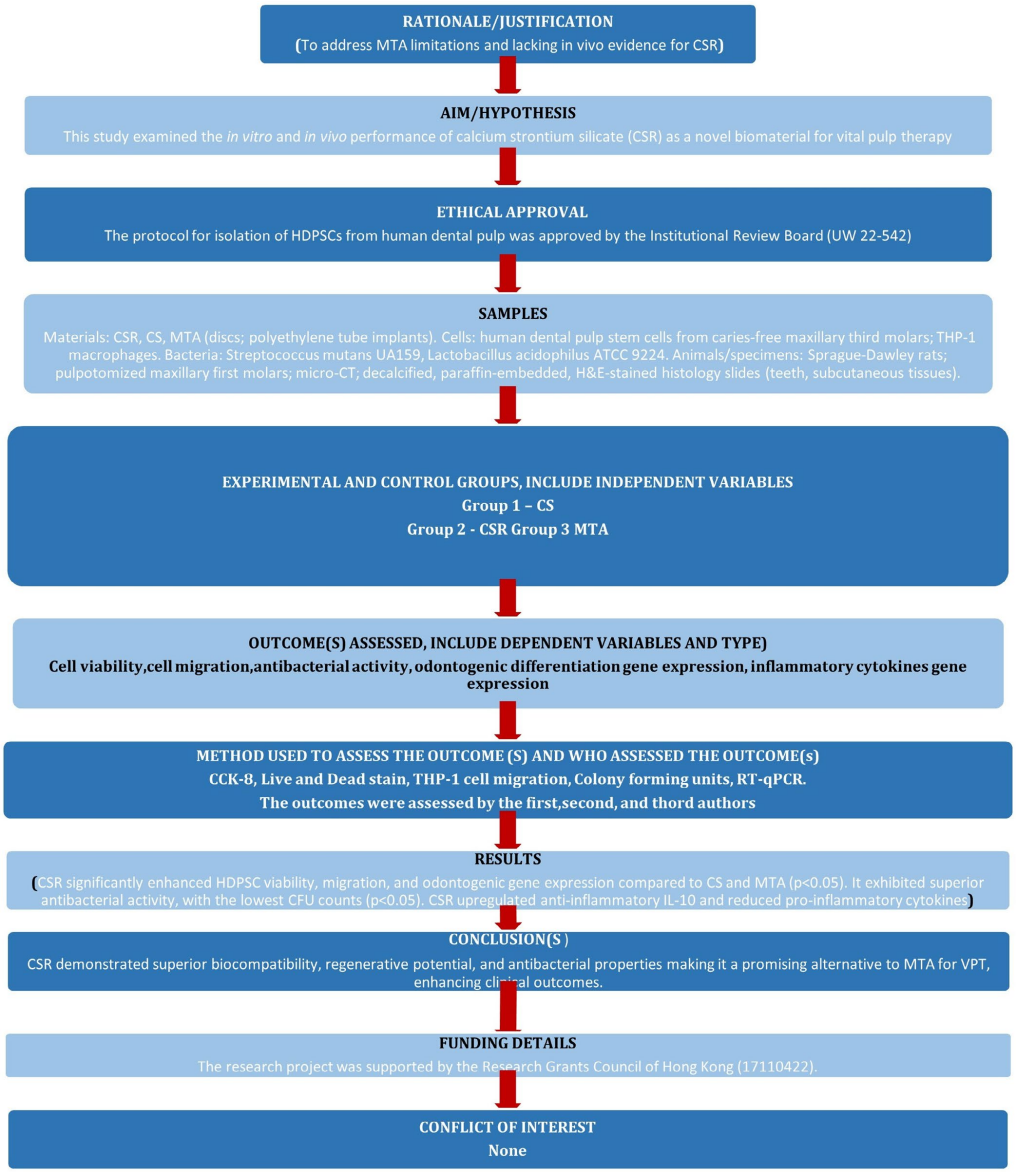
PRILE 2021 flowchart of the experiment.

**FIGURE 2:**
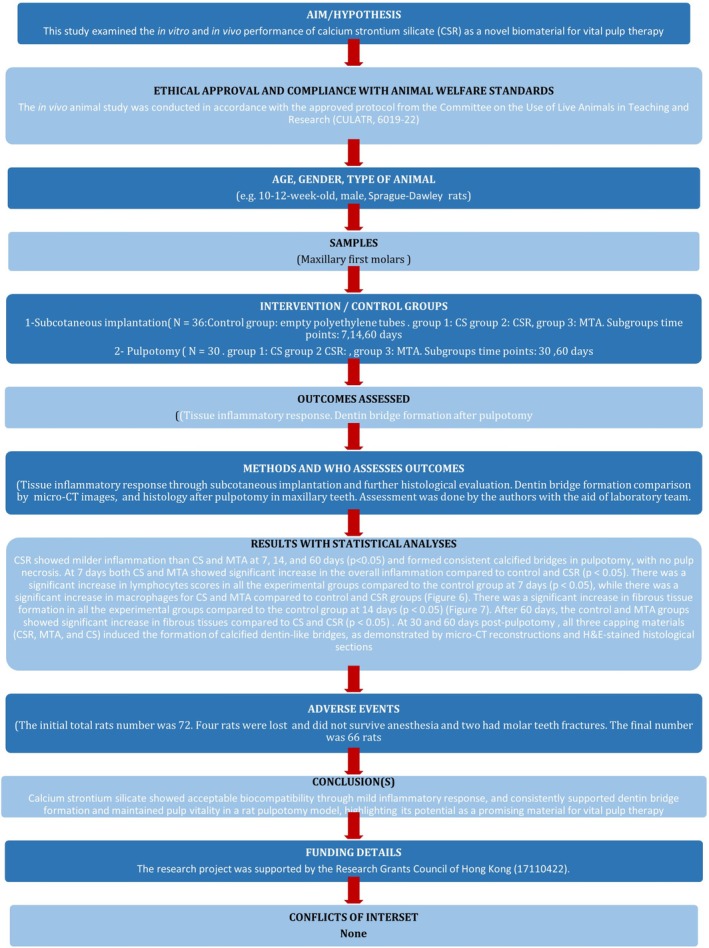
PRIASE 2021 flowchart illustrating the steps involved in conducting the present study.

The manuscript of this laboratory and animal study has been written according to Preferred Reporting Items for Laboratory studies in Endodontology (PRILE) 2021 guidelines (Figure 1), and Preferred Reporting Items for Animal Studies in Endodontology (PRIASE) 2021 guidelines (Figure 2).

### 2.1 Materials Synthesis and Grouping

The synthesis of CS and CSR was conducted according to a previous study. Briefly, Tetraethyl orthosilicate (Si (OC2H5)4; TEOS), obtained from Sigma‐Aldrich (98.0% purity, St. Louis, MO, USA), was mixed with 4 mL of deionized water (DIW) containing 40 μL of HNO3 (69% concentration) (VWR Prolabo Chemicals, Radnor, PA, USA). Following this, Ca (NO3)2 4H2O and Sr. (NO3)2 (Sigma‐Aldrich, 99.0% purity, St. Louis, MO, USA), were incorporated sequentially while maintaining continuous mixing for a duration of 1 h. The molar proportions of Ca, Sr, and Si were kept at 3:1:1.04, respectively. The blend was subsequently heated at 60°C for a period of 24 h and then dried at 120°C for an additional 48 h. The resulting dry gel underwent sintering at 1450°C for 5 h. Ultimately, the derived powders were refined using a ball mill and subjected to sieving. CS was synthesized by adhering to the same methodology while maintaining a Ca:Si molar ratio of 3:1, which was included as a negative control to isolate strontium's effects. MTA (Dentsply, Tulsa Dental, OK, USA) also served as a positive control.

### 2.2 HDPSCs Isolation and Characterization

Isolation of the HDPSCs from human dental pulp was performed according to a protocol approved by the Institutional Review Board of the University of Hong Kong/Hospital Authority Hong Kong West Cluster (UW 22‐542) and informed consent was obtained. Caries‐free maxillary third molars (*n* = 5) were collected from patients aged 25–30 years old at the Oral and Maxillofacial Surgery Clinic. The isolated pulp tissues were processed and then cultivated in α‐minimal essential medium (α‐MEM, GIBCO BRL, Gaithersburg, MD, USA) enhanced with 10% (*v*/*v*) fetal bovine serum (FBS) and 1% (*v*/*v*) penicillin–streptomycin antibiotic solution (ThermoFisher Scientific Inc., Waltham, MA, USA). The cells were maintained in a humidified incubator at 37°C with 5% CO_2_. The medium was changed every 2 days, and the cells were subcultured when they reached 80%–90% confluence. Cells between passages 3 and 5 were used in all the experiments.

For the characterization of HDPSCs, cells were detached with trypsin–EDTA, centrifuged, and resuspended in ice‐cold PBS containing 2% FBS. They were then incubated on ice for 30 min with fluorochrome‐conjugated monoclonal antibodies against CD45, CD73, CD90 and CD105 (Abcam, Cambridge, UK), following manufacturer protocols. After incubation, cells were washed to remove unbound antibodies and immediately analysed by flow cytometry using a fluorescence‐activated cell sorter. The expression profiles of CD45, CD73, CD90 and CD105 were assessed to confirm the mesenchymal stem cell–like phenotype and stemness of the HDPSCs.

### 2.3 CCK‐8 and Live and Dead Stain Cell Viability Assays

To assess the viability of HDPSCs, we compared different control and experimental groups using the CCK‐8 assay. In short, HDPSCs were grown in α‐MEM with 10% fetal bovine serum, penicillin, and streptomycin at 100% humidity, 95% air, 37°C in a 5% CO_2_. Experimental materials were mixed, and extracts were collected under sterile conditions. The HDPSCs were seeded into 96‐wellplate at a density of 10 000 cells per well and incubated for 24 h at 37°C with 5% CO_2_. The cells were then exposed to experimental materials extracts. After 24 h, the cell viability was measured using the CCK‐8 assay kit (Sigma‐Aldrich, St. Louis, MI, USA) according to the manufacturer's instructions. Optical density (OD) was measured at 450 nm using a microplate reader (SpectraMax M2, Molecular Devices, CA, USA). The experiment was performed in triplicate, and the viability was expressed as a percentage relative to the untreated control group after normalization.

The LIVE/DEAD assay (Catalogue No. L3224, Gibco‐Invitrogen, Carlsbad, CA, USA) was used to further evaluate the viability of HDPSCs following the manufacturer's guidelines. The cells were seeded at 3 × 10^4^ cells per well and incubated for 24 h at 37°C and 5% CO_2_. After incubation, the cells were exposed to experimental materials extracts for an additional 24 h. The cells were then rinsed with phosphate‐buffered saline (PBS) and stained with the live and dead staining solution for 1 h. Subsequently, cell images were captured using a fluorescence microscope (Leica DMi8, Leica Microsystems Ltd., USA).

### 2.4 Cell Migration Assay

The trans‐well system used for cell migration assay consisted of two chambers, an upper chamber and a lower chamber, separated by a membrane. The trans‐well inserts (8 μM porous membrane) were placed into the 6‐well plate. The HDPSCs 5 × 10^5^ cell/well were seeded into the upper chamber of each trans‐well, along with the extracts of different materials in ɑ‐MEM. The lower chamber was filled with ɑ‐MEM with 10% of FBS as a migratory stimulus for HDPSCs. The trans‐well plate was incubated at 37°C with 5% CO2 for 24 h, allowing sufficient time for cell migration. After the incubation period, the non‐migrated cells on the upper surface of the membrane were removed using a cotton swab, while the migrated cells on the lower surface of the membrane were fixed using 4% paraformaldehyde and stained with crystal violet to visualize the nuclei using an inverted microscope (Leica DMi8, Leica Microsystems Ltd., USA). The experiment was performed in duplicate using independent biological samples on separate occasions.

### 2.5 Odontogenic Genes Expression Using RT‐qPCR

The HDPSCs were grown in 6‐well plates at a density of 2 × 10^5^ cells per well for 24 h. Following this, the cells were treated with experimental materials for 7 days. Total RNA was extracted from the treated HDPSCs using the RNeasy extraction kit (Qiagen, Hilden, Germany). The concentration of RNA was measured with a spectrophotometer (NanoDrop 2000, Thermo Fisher Scientific), and the RNA was reverse transcribed into complementary DNA (cDNA) using SuperScript Vilo mastermix (Invitrogen, Carlsbad, CA). A quantitative real‐time polymerase chain reaction (qRT‐PCR) was carried out with the ABI Prism 7000 Sequence Detection System (Applied Biosystems, Carlsbad, CA) and Takara SYBR green. The reactions took place at 95°C for 10 min, followed by 40 cycles at 95°C for 15 s, and then 60°C for 1 min. Primers (Table 1) for the HDPSCs genes, including alkaline phosphatase (ALP), dentine sialophosphoprotein (DSPP), dentine matrix protein 1 (DMP‐1), runt‐related transcription factor 2 (RUNX‐2) and the housekeeping gene glyceraldehyde‐3‐phosphate dehydrogenase (GAPDH), were utilized. For data analysis, the StepOne software v2.0.2 (Applied Biosystems) determined the expression levels of target genes in the samples compared to the control samples using the comparative cycle threshold method (ΔΔCT). Relative gene expression was calculated using the 2^−∆∆Ct^ method, with all reactions performed in duplicate using independent biological samples at different occasions.

**TABLE 1:** Primer's sequence used for RT‐qPCR.

Gene	Primers sequence
*ALP*	F: ACGTGGCTAAGAATGTCATC R: CTGGTAGGCGATGTCCTTA
*DSPP*	F: ATATTGAGGGCTGGAATGGGGA R: TTTGTGGCTCCAGCATTGTCA
*DMP‐1*	F: GAGCAGTGAGTCATCAGAAGGC R: GAGAAGCCACCAGCTAGCCTAT
*RUNX2*	F: ACTCTACCACCCCGCTGTC R: CAGAGGTGGCAGTGTCATCA
*TNF*α	F: CTGCACTTTGGAGTGATCGG R: TCAGCTTGAGGGTTTGCTAC
*IL‐1β*	F: GCCCTAAACAGATGAAGTGCTC R: GAGATTCGTAGCTGGATGCC
*IL‐10*	F: GGGCACCCAGTCTGAGAACA R: GACAAGGCTTGGCAACCCAG
*GAPDH*	F: TGGCACCCAGCACAATGAA R: CTAAGTCATAGTCCGCCTAGAAGCA

### 2.6 Antibacterial Activity Assessment

*S. mutans* UA159 (ATCC 700610) and *L. acidophilus* (ATCC 9224) were cultured in brain heart infusion (BHI) broth and maintained at 37°C in an anaerobic environment (85% N_2_, 10% H_2_, 5% CO_2_). The bacterial suspension was adjusted to a concentration of 10^7^ CFU/mL using the McFarland spectrophotometric method, with measurements taken at an optical density of 600 nm (OD600nm).

### 2.7 Evaluation of Colony‐Forming Units (CFU)

The fabricated discs of various experimental materials were positioned in 12‐well plates and incubated with 1 mL of 1:1 bacterial suspension (10^7^ CFU/mL) under anaerobic conditions for 24 h. Following incubation, the discs were suspended in 1 mL of PBS. The suspensions were vortexed for 1 min to disperse the biofilm, serially diluted, and spiral plated onto selective agar plates (Mitis Salivarius and Rogosa agar plates for *S. mutans* and *L. acidophilus* , respectively) and incubated anaerobically for 48 h. The colonies were counted, and the CFU was reported as the mean ± standard deviation of log_10_ CFU/mL.

### 2.8 Inflammatory Cytokines Gene Expression

The THP‐1 monocytes were cultured in RPMI 1640 medium (Gibco, Life Technologies, NY, USA) supplemented with 10% FBS (Sigma Aldrich St. Louis, MO, USA) and 1% penicillin/streptomycin solution. To promote monocyte to macrophage differentiation, 3 × 10^6^ cells were seeded with media containing 150 nM of Phorbol‐12‐myristate‐13‐acetat e (PMA) (Sigma Aldrich St. Louis, MO, USA) for 24 h. Afterward, the cells were replenished with fresh PMA‐free media and cultured for an additional 72 h. To induce inflammatory conditions, THP‐1 macrophages were stimulated with 100 ng of lipopolysaccharide (LPS) ( *E. coli* 055: B5 invivoGen, San Diego, USA) for 24 h. Following LPS stimulation, the cells were treated with materials extracts for 24 h, and the expression of pro‐inflammatory cytokines IL‐1β and TNF‐α, as well as the anti‐inflammatory cytokine IL‐10 was quantified in duplicate from independent biological samples in independent experiments using qRT‐PCR as described above. The sequences of the primers are described in Table 1.

### 2.9 In Vivo Experiments

The in vivo animal study was conducted in accordance with the approved protocol from the Committee on the Use of Live Animals in Teaching and Research (CULATR, 6019‐22). Sprague–Dawley (SD) rats, aged 10–12 weeks and weighing between 500 and 550 g, were utilized for this study. The animals were housed in a controlled environment with a 12‐h light/dark cycle, constant temperature (22°C ± 2°C) and relative humidity (50%). They were given ad libitum access to food and water throughout the study.

### 2.10 In Vivo Inflammatory Response

A total of 36 Sprague–Dawley (SD) male rats were divided into four experimental and control groups with 12 rats each. Each group was further subdivided into four subgroups based on the time points for analysis: 7, 14 and 60 days. Sterile polyethylene tubes, with a diameter of 1.5 mm and a length of 10 mm were utilized for the study. The tubes were filled with experimental materials inside a cell culture hood under aseptic conditions. Empty tubes served as the control. The rats were anaesthetised using isoflurane inhalation. This allowed for the assessment of the potential inflammatory effects without the interference of ketamine and xylazine, which have immunosuppressive properties. The dorsal surface of the rats was shaved and disinfected with betadine, then a 2 cm incision was made. Two polyethylene tubes were implanted subcutaneously, one on each side of the incision. The incision site was then suture closed. At the predetermined time points (7, 14 and 60 days), the rats were euthanized, and the implanted tubes were excised along with the surrounding tissues and were carefully fixed in 4% paraformaldehyde solution for a period of 24 h. Following fixation, the samples underwent paraffin embedding. Using a microtome, the embedded samples were sliced into slides with a thickness of approximately 7 μm. The prepared slides were subjected to Haematoxylin and Eosin (H&E) staining. Inflammation was evaluated within standardized regions of interest located directly beneath the capping materials in the polyethylene tubes (as indicated by asterisks) by two independent, blinded specialists. Inter‐rater reliability was calculated using Cohen's Kappa statistic (SPSS v.28), with *κ* > 0.61–0.80 considered strong agreement. To assess the inflammatory response, inflammatory cells such as neutrophils, lymphocytes, macrophages and plasma cells were counted and given a score based on the following criteria:

1. Absent, no inflammation
2. Mild, cells sparse or in reduced clusters.
3. Moderate cells were present but did not dominate the microscopic field.
4. Intense, cells were present in the form of an infiltrate.

The data obtained from the cell counting analysis was further subjected to statistical analysis to determine any significant differences between the control group and the experimental groups. Additionally, the slices were examined for the presence of fibroblasts, fibrous tissues and blood vessels. The overall inflammation score was also calculated.

### 2.11 Pulpotomy in Rats' Maxillary First Molars

Pulpotomy procedures were performed on the right and left maxillary first molars of 30 SD rats, yielding a total of 60 teeth, which were subsequently allocated to three primary experimental groups: CS, CSR and MTA (*n* = 20 teeth). Each primary group was further subdivided into two subgroups, with analysis time points set at 30 and 60 days (*n* = 10 teeth). Prior to the pulpotomy procedure, the rats were anaesthetised using a combination of ketamine (80 mg/kg) and xylazine (10 mg/kg), administered through intraperitoneal injection. The maxillary first molars and surrounding soft tissue were disinfected using chlorhexidine. The pulpotomy was performed under proper isolation conditions. An access cavity was created on the maxillary first molar using a size 0.5 carbide bur. The pulp chamber was then exposed, and the residual pulp chamber tissues were meticulously excavated. Haemostasis was achieved through irrigation with 2.50% sodium hypochlorite (Clorox Corp., Oakland, USA) and the pulp chamber space was dried with absorbent paper points. The experimental materials were mixed and applied directly to the exposed pulp tissue within the access cavity. The access cavity was sealed using resin modified glass ionomer cement (Fuji II LC, GC America, IL, USA) to ensure a proper barrier against any potential contamination. At the predetermined time points, the rats were euthanized and the maxilla at the teeth site was excised for further processing. High‐resolution micro‐computed tomography (micro‐CT) scans were obtained to assess the formation of calcified dentine bridge. Afterwards, the extracted teeth underwent a decalcification process using 12% ethylenediaminetetraacetic acid (EDTA, Sigma‐Aldrich, St. Louis, MO, USA) with constant agitation for 96 h. Subsequently, the decalcified teeth were processed and embedded in paraffin wax. Using a microtome, 6‐μm‐thick sections were prepared from the paraffin‐embedded teeth. The sections were then stained with H&E to visualize cellular components and tissue structures. The stained histological sections were examined under a microscope to assess the pulp tissue response and the quality of the calcified bridge formation.

The thickness of dentine bridge was measured on the digitized histological sections and the reconstructed micro‐CT images using Fiji (ImageJ 2.17.0, MD, USA). After importing the images into Fiji, the pixel‐to‐micrometre ratio was calibrated by drawing a line along the microscope scale bar and entering the known distance. For each section, three measurements of dentine bridge thickness were obtained using the straight‐line tool, with each line drawn perpendicular to the long axis of the calcified bridge. The average of these three measurements represented the dentine bridge thickness for that section.

### 2.12 Statistical Analysis

The statistical package for the social sciences (SPSS) version 28 (IBM, Armonk, NY, USA) was used for data analysis. One‐way ANOVA was used to analyse the antibacterial activity, RT‐qPCR and dentine bridge thickness results, while a non‐parametric test was used to analyse cell viability and in vivo inflammatory response, with the significance level set at (*p* < 0.05).

## 3 Results

### 3.1 Characterization of HDPSCs

The flow cytometry analysis revealed high expression of mesenchymal markers in DPSCs, with 100% positivity for CD73, 99.4% for CD90 and 58.8% for CD105, alongside negligible CD45 expression (0.336%) (Figure S1).

### 3.2 Cell Viability and Live and Dead Staining Assay

**FIGURE 3:**
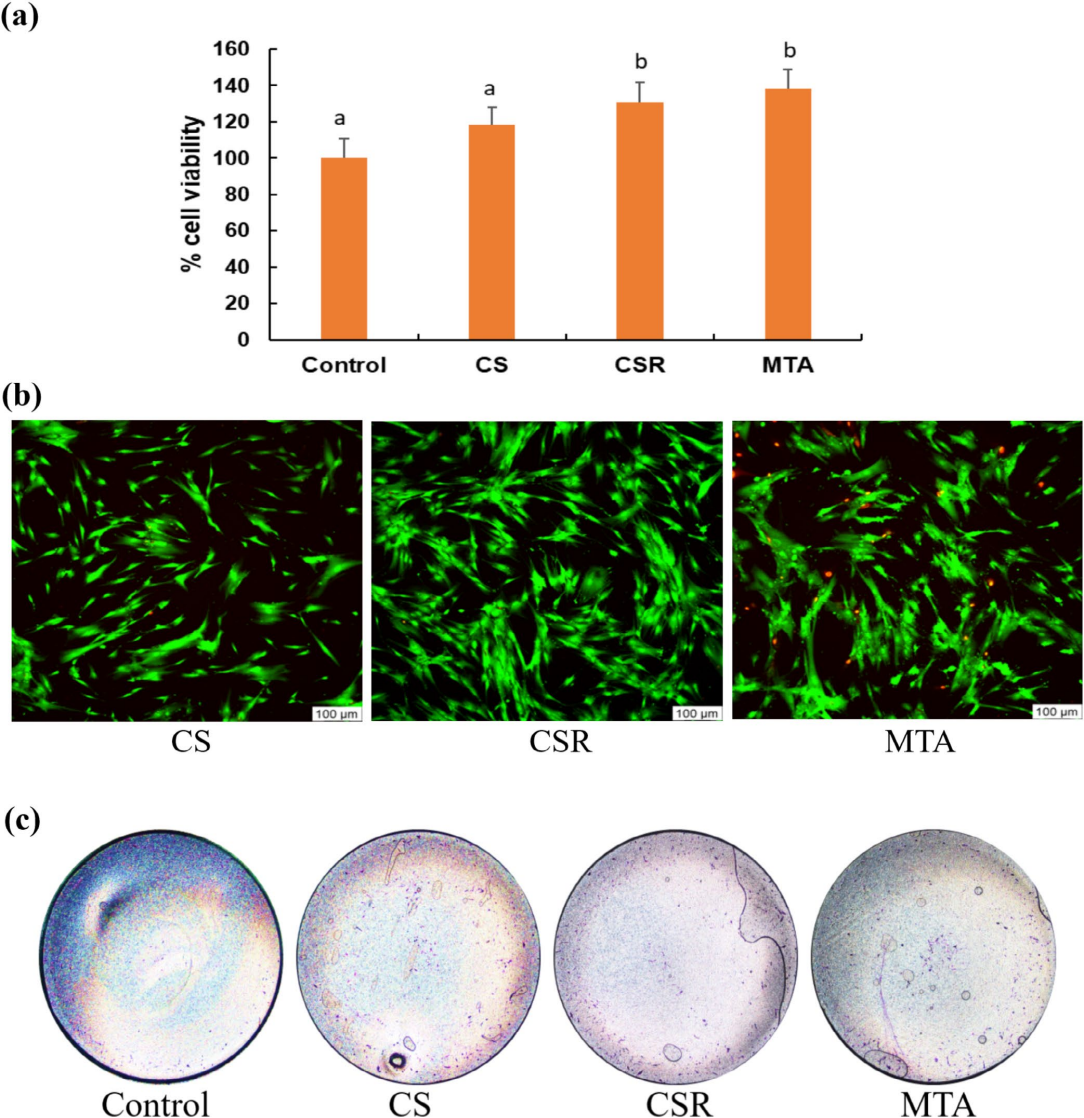
(a) Average cell viability of the test materials normalized to control. Different lower‐case letters indicate significant differences (*p* < 0.05). (b) Live and dead stain of DPSCs. Viable cells represented by green while dead cells represented by red. Scale bars = 100 μm. (c) Migrated cells on the lower surface of the trans‐well membrane stained with crystal violet.

The viability of HDPSCs in response to CSR and MTA was significantly higher than that of the control (*p* < 0.05) (Figure 3a). Moreover, the live and dead stain results demonstrated prevailing live cells (represented by the green) in all the tested materials, suggesting optimal biocompatibility of the tested groups (Figure 3b).

### 3.3 Cell Migration Assay

The DPSCs treated with materials extracts showed remarkable cell migration toward the serum medium compared to the control group (Figure 3c).

### 3.4 Odontogenic Differentiation Gene Expression

**FIGURE 4:**
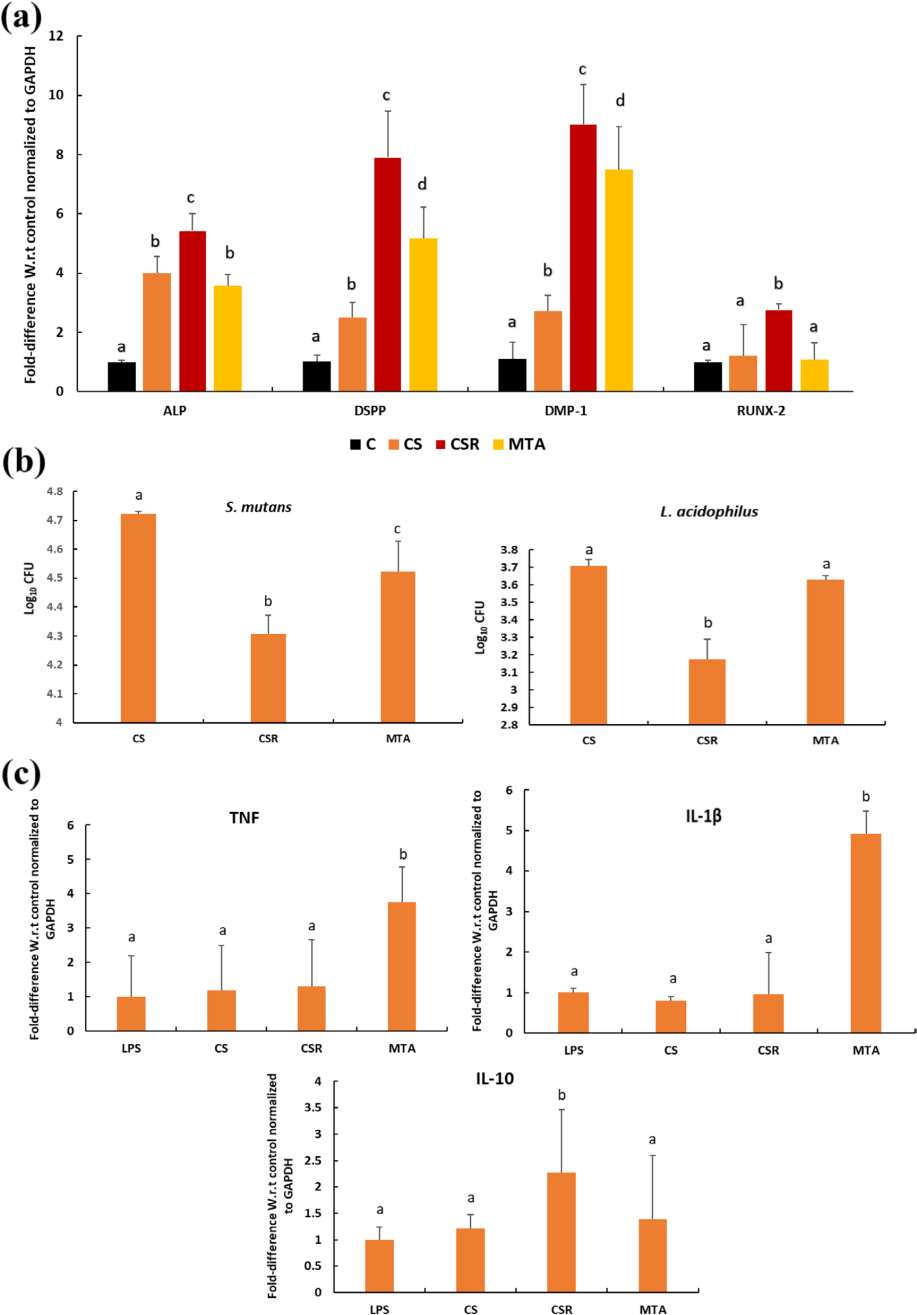
(a) Fold change in the gene expression of ALP, DSPP, DMP‐1, and RUNX2 among the test groups. Different lower‐case letters indicate significant differences (*p* < 0.05). (b) Log_10_ CFU of *S. mutans* and *L. acidophilus*. Different lower‐case letters indicate significant differences (*p* < 0.05). (c) Fold change in the gene expression of the inflammatory markers IL‐1β, IL‐10 and TNF‐α. Different lower‐case letters indicate significant differences (*p* < 0.05).

The results of RT‐qPCR showed significant upregulation of ALP, DSPP, DMP‐1, and RUNX2 in CSR after 7 days of culture compared to CS and MTA test groups (*p* < 0.05). The expression of DSPP and DMP‐1 was significantly higher in MTA than CS group (*p* < 0.05) (Figure 4a).

### 3.5 Antibacterial Activity

The CFU count for *S. mutans* was significantly different in all the tested groups (*p* < 0.05). The CSR group showed the lowest CFU count followed by MTA and CS. While for *L. acidophilus* , CSR displayed significantly lower CFU compared to CS and MTA (*p* < 0.05) (Figure 4b).

### 3.6 Inflammatory Cytokines Gene Expression

The expression of the pro‐inflammatory markers IL‐1β and TNF‐α was significantly higher in MTA than CS and CSR (*p* < 0.05). Whereas the expression of the anti‐inflammatory marker IL‐10 was significantly higher in CSR compared to CS and MTA (*p* < 0.05) (Figure 4c).

### 3.7 In Vivo Inflammatory Response

**FIGURE 5:**
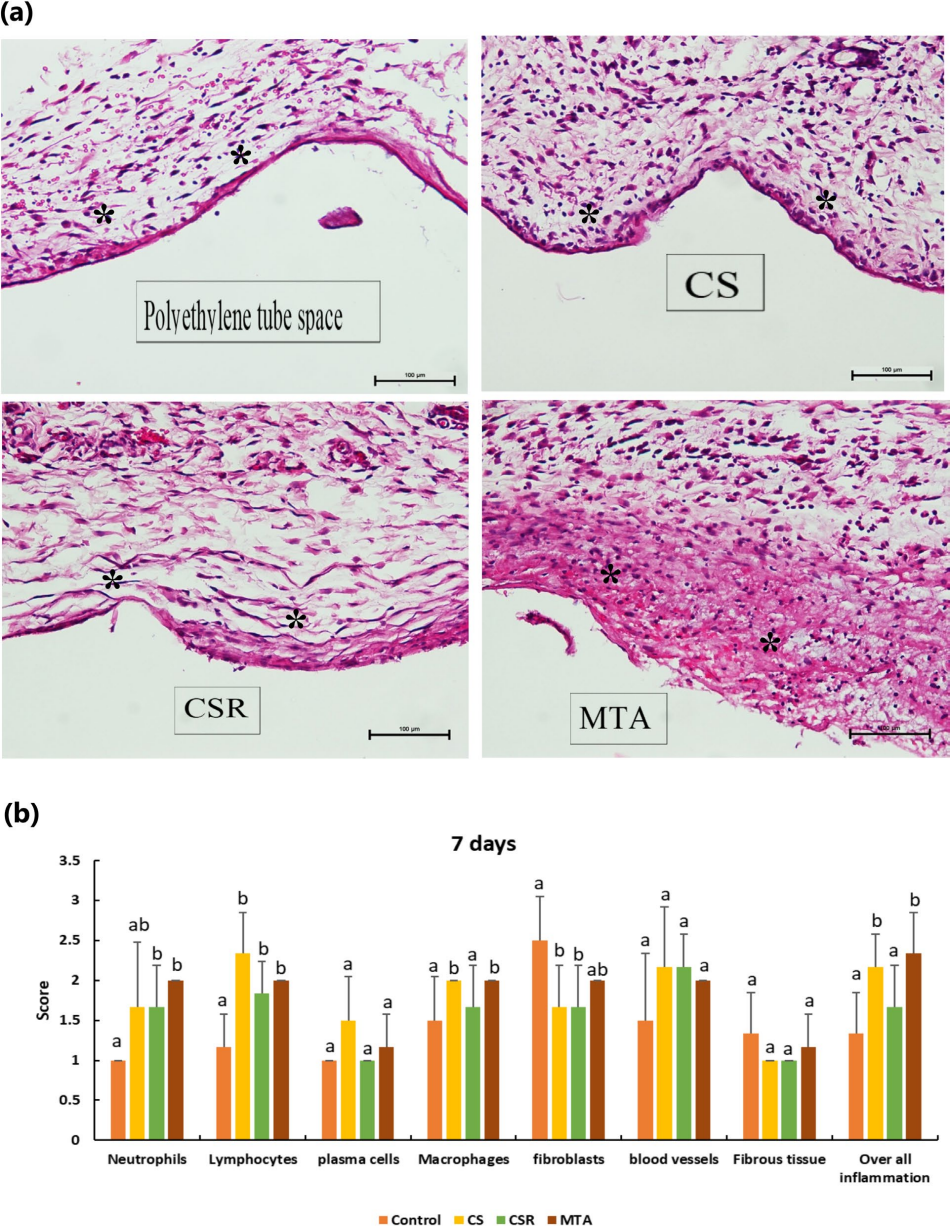
(a) H&E‐stained sections showing connective tissue bordering the polyethylene tube space as a control and polyethylene tube opening containing experimental materials. A fibrous capsule is present at the interface with inflammatory cells dispersed within the adjacent connective tissue. Standardized regions of interest for inflammation grading are indicated by asterisks; evaluations were performed by a single examiner blinded to group allocation. Scale bar = 100 μm. (b) Results of the scoring among the experimental groups evaluating the different cellular inflammatory responses after 7 days. Different lower‐case letters indicate significant differences within each variable cell (*p* < 0.05).

**FIGURE 6:**
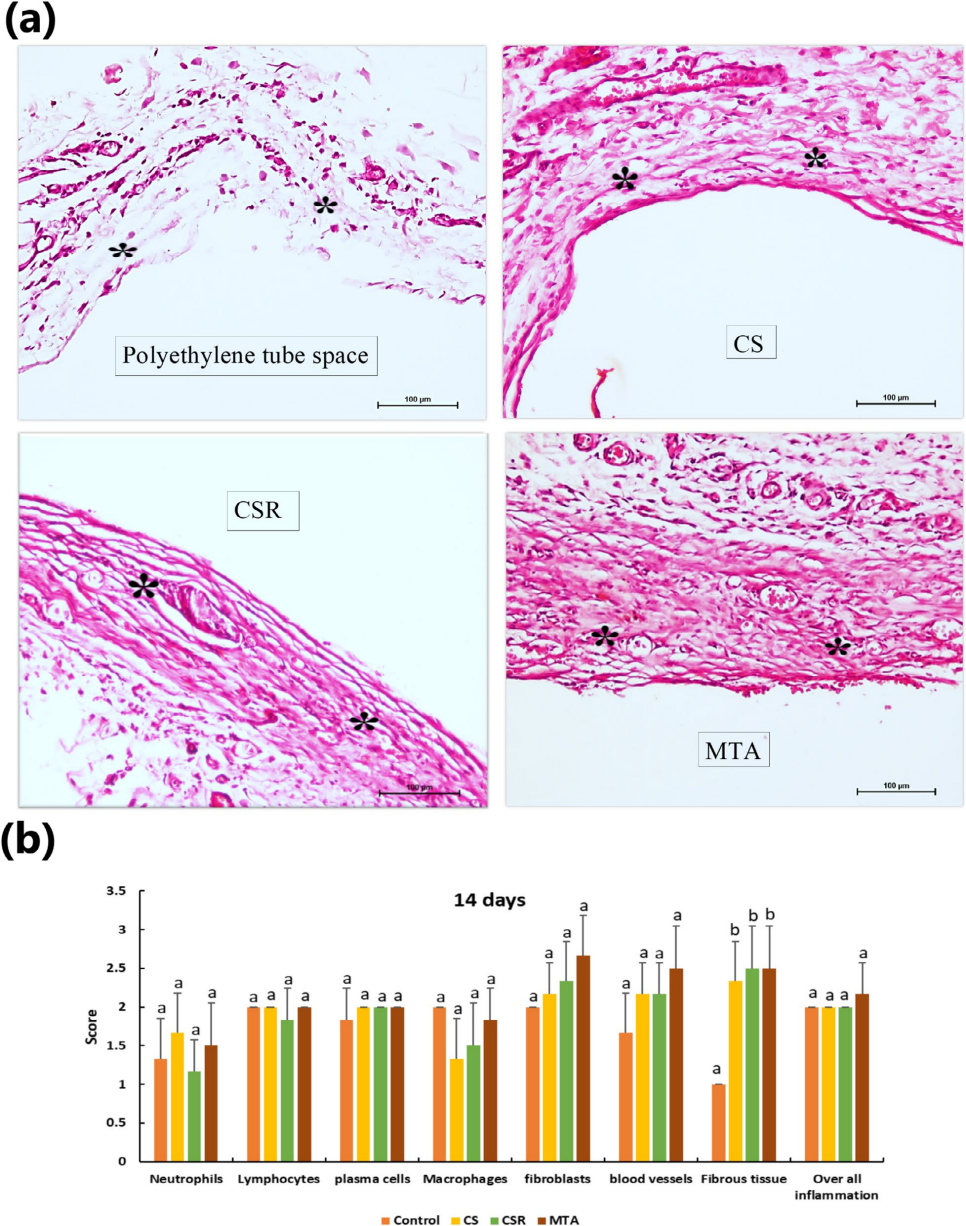
(a) H&E section at the interface with the polyethylene tube space and polyethylene tube opening containing experimental materials. Fibrous capsule and surrounding connective tissue contain scattered inflammatory cells. Asterisks indicate the standardized regions of interest used for inflammatory scoring. Scale bar = 100 μm. (b) Results of the scoring among the experimental groups evaluating the different cellular inflammatory responses after 14 days. Different lower‐case letters indicate significant differences (*p* < 0.05) in each variable cell.

**FIGURE 7:**
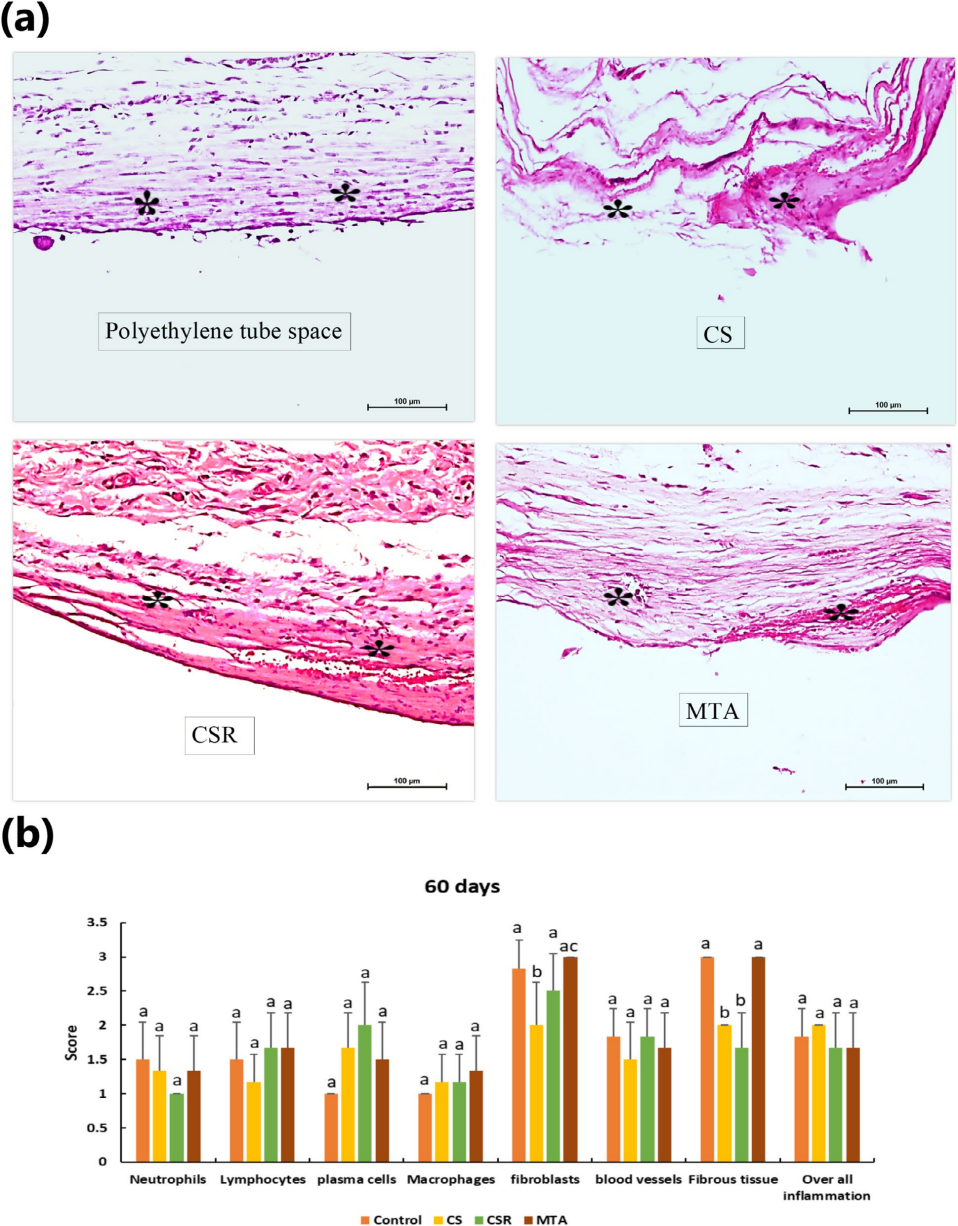
(a) H&E section at the interface with the polyethylene tube space and polyethylene tube opening containing experimental materials. Fibrous capsule and surrounding connective tissue contain scattered inflammatory cells. Asterisks indicate the standardized regions of interest used for inflammatory scoring. Scale bar = 100 μm. (b) Results of the scoring among the experimental groups evaluating the different cellular inflammatory responses after 60 days. Different lower‐case letters indicate significant differences (*p* < 0.05) in each variable cell.

Histological scoring demonstrated strong inter‐rater agreement (Cohen's κ = 0.7, 95% CI). The overall evaluation of the in vivo inflammatory response showed that mild to moderate inflammation persisted in all the tested groups with no signs of necrosis observed. At 7 days both CS and MTA showed significant increase in the overall inflammation compared to control and CSR (*p* < 0.05). There was a significant increase in lymphocytes scores in all the experimental groups compared to the control group at 7 days (*p* < 0.05), while there was a significant increase in macrophages for CS and MTA compared to control and CSR groups (Figure 5). There was a significant increase in fibrous tissue formation in all the experimental groups compared to the control group at 14 days (*p* < 0.05) (Figure 6). After 60 days, the control and MTA groups showed significant increase in fibrous tissues compared to CS and CSR (*p* < 0.05) (Figure 7).

### 3.8 Pulpotomy in Rat Molars

**FIGURE 8:**
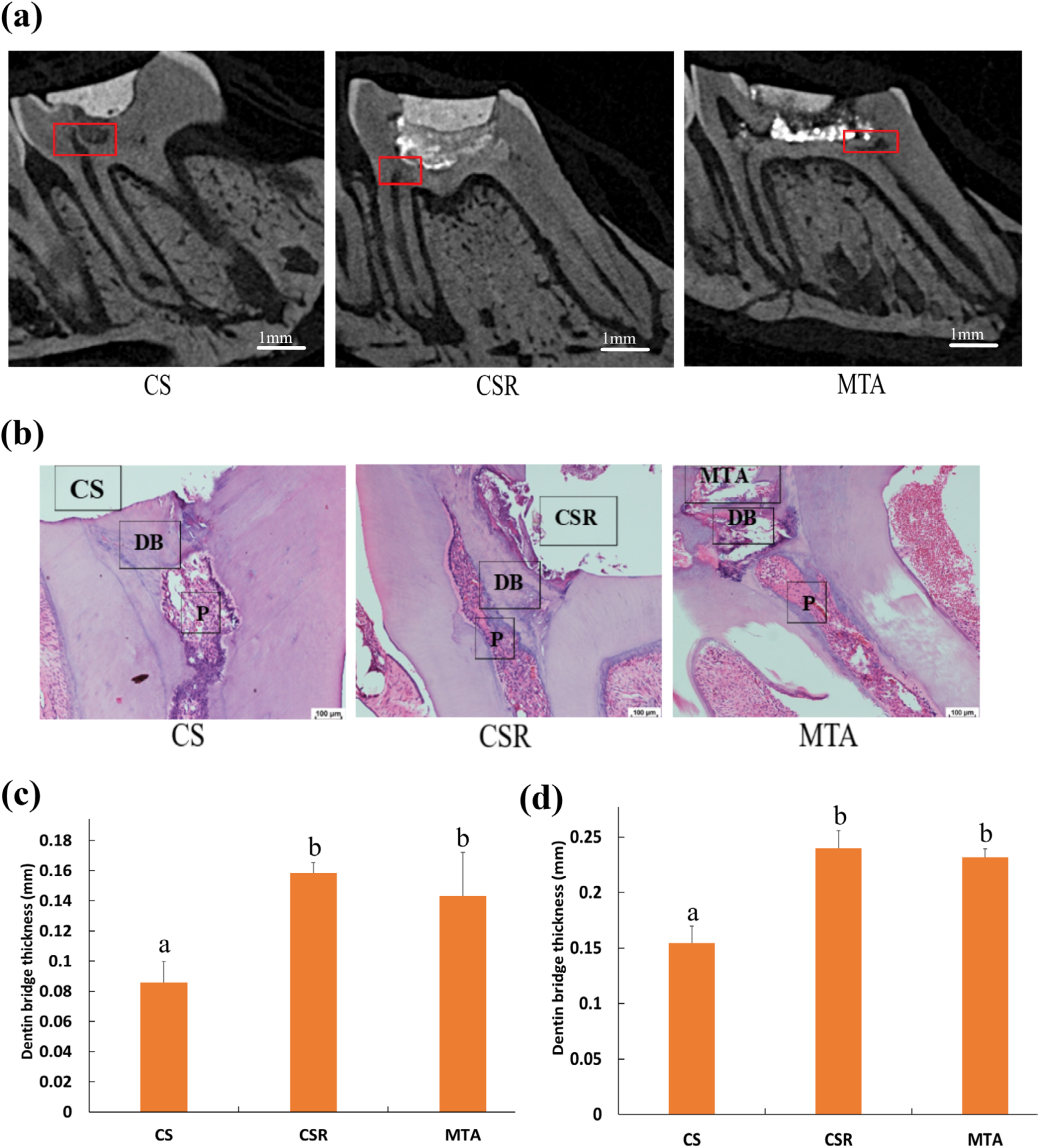
(a) Representative micro‐CT sections of pulpotomy‐treated rat maxillary molars at 30 days in the CS, CSR, and MTA groups. Red boxes delineate the region of interest at the pulp/capping materials interface, highlighting the formed calcified bridge. Scale bars = 1 mm. (b) Representative H&E‐stained sections of pulpotomy‐treated rat maxillary molars at 60 days. Boxed annotations indicate the capping materials, the dentine bridge (DB) at the exposure site, and the underlying pulp (P). The dentine bridge appears as an eosinophilic mineralized band spanning the cut dentine margins. Scale bars = 100 μm. (c) Average values of dentine bridge thickness in mm after 30 days, while (d) represents average values of dentine bridge thickness in mm after 60 days. Different lower‐case letters indicate significant differences (*p* < 0.05).

At 30 and 60 days post‐pulpotomy (Figure 8a,b), all three capping materials (CSR, MTA, and CS) induced the formation of calcified dentine‐like bridges, as demonstrated by micro‐CT reconstructions and H&E‐stained histological sections. The pulp subjacent to the formed bridge preserved normal architecture, including intact vasculature and an organized odontoblastic layer, without histological evidence of necrosis or degenerative change at either time point, indicating the potential of the tested materials in developing dentine bridge and preserving the vitality of the pulp. The formed dentine bridge was significantly thicker in CSR and MTA compared to CS (*p* < 0.05) (Figure 8c,d).

## 4 Discussion

This study evaluated the effectiveness of CSR as a novel biomaterial for VPT, comparing its performance with CS and MTA. Since CSR demonstrated higher upregulation of odontogenic differentiation gene such as ALP, DSPP DMP‐1 and RUNX2, along with greater inhibition of *S. mutans* and *L. acidophilus* compared to CS and MTA, and exhibited lower expression of pro‐inflammatory markers IL‐1β, TNF‐α, as well as higher expression of the anti‐inflammatory cytokine IL‐10 compared to MTA, the first null hypothesis has to be rejected. Conversely, CSR elicited lower scores and reduced inflammatory cell density compared to CS and MTA, and the dentine bridge was thicker in CSR and MTA compared to CS. Therefore, the second null hypothesis also has to be rejected.

In a previous study, CSR possessed enhanced physicochemical properties, including a significantly shorter setting time than MTA. This is particularly important for streamlining dental procedures (Abdalla et al. 2022). In the present study, CSR offered superior biocompatibility, greater odontogenic potential, enhanced antibacterial effects, and improved immunomodulatory properties when compared to MTA and CS. Additionally, we examined the tissue inflammatory response following subcutaneous implantation of the tested materials. We also investigated CSR's ability to promote the formation of a calcified bridge in an in vivo rat model, a widely accepted method for assessing pulpotomy outcomes, due to its reliability and relevance to endodontic research (Aubeux et al. 2021; Dammaschke 2010). Together, these findings demonstrate that CSR outperforms MTA overall, making it a promising alternative for VPT, supporting the rejection of the null hypothesis tested.

The in vitro cell viability assessment demonstrated that CSR and MTA significantly enhanced the viability of HDPSCs compared to respective controls. This was evidenced by CCK‐8 and live/dead staining assays, both of which indicated a predominance of viable cells. These findings are consistent with previous studies highlighting the beneficial role of Sr. in promoting pulp cell survival and proliferation (Abdalla et al. 2024; Oudadesse et al. 2011). The high biocompatibility exhibited by CSR underscores its potential suitability for VPT, where the maintenance of pulp cell vitality is crucial for tissue repair and regeneration. Strontium mimics calcium and binds to CSR on cell membranes, triggering intracellular signalling cascades (e.g., MAPK/ERK) that promote cell survival and proliferation (Brown and MacLeod 2001). The enhanced migratory capacity is crucial for recruiting regenerative cells to the site of pulp injury, facilitating tissue repair (Howard et al. 2010). Our findings demonstrated that CSR significantly promoted HDPSCs migration. Similar findings were reported by Zhou et al. (2024), who noted that Sr‐containing bioceramic sealer promoted cell migration, which was attributed to the active effect of Sr. on cellular behaviour. MTA's delayed Ca^2+^ release and transient cytotoxicity at high pH reduce its ability to induce cell migration compared to CSR (Prati and Gandolfi 2015).

The early upregulation of the odontogenic genes (ALP, DSPP, DMP‐1, RUNX2) is critical for initiating dentinogenesis, as ALP and RUNX2 regulate early mineralization, while DSPP and DMP‐1 are essential for dentine matrix formation (Mortada and Mortada 2018). This early upregulation aligns with findings by Huang et al. (Huang et al. 2016), who reported that Sr. enhanced odontogenic differentiation in HDPSCs. This rapid induction of these genes by CSR suggests its potential to accelerate reparative dentine formation, a key factor in the success of VPT, as supported by clinical studies emphasizing early dentine bridge formation.

CSR exhibited significantly lower colony‐forming unit (CFU) counts for *S. mutans* and *L. acidophilus* compared to CS and MTA (*p* < 0.05). These bacterial strains are prevalent in deep carious lesions, where they contribute to pulp inflammation and treatment failure (Jung et al. 2019; Bjørndal et al. 2019). Although the precise antibacterial mechanism of Sr. is unclear, Sr. may disrupt metabolic processes by inhibiting adenosine triphosphate (ATP) synthesis or by producing reactive oxygen species, which can cause severe damage to cell membranes (Abdalla et al. 2023; Thongsri et al. 2024; Alshammari et al. 2021).

RT‐qPCR analysis revealed that CSR and CS significantly reduced pro‐inflammatory cytokines (IL‐1β, TNF‐α) while upregulating anti‐inflammatory IL‐10 in THP‐1 macrophages compared to MTA. IL‐1β and TNF‐α are key mediators of inflammation, driving tissue damage and chronic pulpitis if overexpressed, while IL‐10 promotes tissue repair by modulating immune responses (Abdalla et al. 2024; Abuarqoub et al. 2022). CSR's ability to suppress IL‐1β and TNF‐α while enhancing IL‐10 suggests a shift toward an anti‐inflammatory macrophage phenotype, which is critical for creating a regenerative microenvironment (You et al. 2022). Strontium has been found to potentially regulate the macrophage responses, which aligns with other studies investigating strontium's immunomodulatory effects (Zhang et al. 2016; Lee et al. 2016; Lu et al. 2019).

To further assess the biocompatibility of the experimental materials in this study, we investigated the inflammatory response following subcutaneous implantation of the test materials in Sprague–Dawley rats. The in vivo inflammatory response showed mild to moderate inflammation across all groups. These inflammatory cells are critical indicators of tissue response, with neutrophils and macrophages primarily involved in driving acute inflammation, while lymphocytes and plasma cells signify ongoing chronic immune activation (Delfino et al. 2020). Our findings are in line with previous studies on Sr‐containing biomaterials, which demonstrated mild inflammatory effects in vivo (Pinheiro et al. 2018; Pelepenko et al. 2022). The use of isoflurane anaesthesia avoided the immunosuppressive effects of ketamine and xylazine, ensuring reliable inflammatory data (Anderson et al. 2014).

Our study showed positive regenerative outcomes using CSR in a healthy pulp exposure model, these findings are situated within the broader clinical context of VPT. In reversible pulpitis with carious exposure, the inflamed coronal pulp is removed during pulpotomy until the canal orifices, exposing presumably healthy, vital radicular pulp for material capping (American Association of Endodontists 2021; Ricucci et al. 2019). This ensures the biomaterial interfaces with non‐inflamed tissue, promoting odontoblast differentiation and tertiary dentine formation without the confounding effects of persistent inflammation, closely simulating our mechanical exposure model in healthy teeth (Kahler and Rossi‐Fedele 2023). Furthermore, the initial in vivo testing of CSR in less variable conditions prioritizes establishing baseline biocompatibility and efficacy, which is common in preclinical endodontic research where healthy animal models precede caries‐ or lipopolysaccharide‐induced inflammation models to isolate material‐specific effects (Xuan et al. 2023; Aubeux et al. 2021). For instance, early evaluations of MTA utilized similar healthy exposures to confirm regenerative potential before advancing to diseased states (Ford et al. 1996). In irreversible pulpitis, where radicular inflammation may persist, further studies in inflamed models are warranted to expand CSR's applicability; nonetheless, our findings provide a robust foundation for such investigations, aligning with progressive VPT research paradigms. We chose comparative biomaterial controls to align with ethical standards and established preclinical protocols rather than including an untreated group. This precludes direct assessment of baseline healing responses in the absence of any intervention and should be considered when interpreting the relative efficacy of the tested materials.

Micro‐CT imaging and H&E staining of rat molar pulpotomy sites demonstrated that CS, CSR and MTA induced well‐developed, continuous calcified bridges at both 30—and 60—days post‐treatment. CSR, in particular, showed consistent dentine bridge formation with no evidence of pulp necrosis, comparable to the outcomes observed with MTA, which is considered the gold standard pulp capping material. The formation of a continuous calcified bridge is critical for sealing and protecting the pulp from microbial infiltration and preserving pulp vitality (Murray and García‐Godoy 2006). These findings corroborate previous studies reporting robust dentine bridge formation and sustained pulp vitality in rat pulpotomy models treated with bioceramic materials (Minic et al. 2021; Tran et al. 2019).

The study has some limitations that need to be acknowledged. First, we did not use a pulpitis‐ induced rat model; instead, pulpotomies were performed on healthy teeth, however, this model does not fully replicate inflammatory clinical conditions, and that future studies using disease‐relevant models would be valuable to extend the translational applicability of the findings. Second, while the follow‐up periods of up to 60 days provided valuable insight into short‐ to mid‐term healing, longer observation periods are needed to evaluate the durability and stability of the reparative tissue over time. Third, although the in vivo rat model offers important preclinical data, it does not completely mimic human dental anatomy, immune responses or the complex oral environment. Therefore, the findings from the animal study require further validation through well‐designed clinical trials in human subjects before they can be recommended for routine clinical application. Furthermore, this study primarily focused on histological and radiographic outcomes, future work should incorporate functional assessments such as pulp sensibility and mechanical testing of the regenerated tissue.

## 5 Conclusions

Our study demonstrated that CSR exhibited enhanced biocompatibility, odontogenic differentiation and possessed strong antibacterial and immunomodulatory properties compared to MTA. Calcium strontium silicate consistently supported dentine bridge formation and maintained pulp vitality in a rat pulpotomy model, highlighting its potential as a promising material for vital pulp therapy. However, further research using disease‐relevant models, extended follow‐up periods and clinical trials in humans is necessary to fully establish the efficacy, safety and long‐term performance of CSR in clinical practice.

## Author Contributions

**Mohamed Mahmoud Abdalla:** conceptualization, analysis, experimental procedures, writing, review. **Vidhyashree Rajasekar:** experimental procedures, writing. **Heba Ahmed:** experimental procedures, analysis. **Mengyu Huang:** analysis, experimental procedures. **Cynthia Kar Yung Yiu:** critical review, and editing (lead).

## Funding

This work was supported by General Research Fund, Research Grants Council of Hong Kong, 17110422.

## Conflicts of Interest

The authors declare no conflicts of interest.

## Supporting information


**Figure S1:** presents the flow cytometric characterization of the isolated HDPSCs which demonstrated high expression of mesenchymal markers in DPSCs, with 100% positivity for CD73, 99.4% for CD90 and 58.8% for CD105, alongside negligible CD45 expression (0.336%).

## Data Availability

The data that support the findings of this study are available from the corresponding author upon reasonable request.
